# Description of social contacts among student cases of pandemic influenza during the containment phase, Melbourne, Australia, 2009

**DOI:** 10.5365/wpsar.2018.9.5.003

**Published:** 2018-10-01

**Authors:** Caroline van Gemert, Emma S McBryde, Isabel Bergeri, Rachel Sacks-Davis, Hassan Vally, Tim Spelman, Brett Sutton, Margaret Hellard

**Affiliations:** a Burnet Institute, Melbourne, Victoria, Australia.; b Department of Epidemiology and Preventative Medicine, Monash University, Alfred Hospital, Commercial Road, Melbourne, Victoria, Australia.; c Centre for Biosecurity in Tropical Infectious Diseases, James Cook University.; d Department of Medicine, University of Melbourne.; e School of Psychology and Public Health, La Trobe University, Melbourne.; f Victorian Department of Health and Human Services.

## Abstract

**Introduction:**

Students comprised the majority of early cases of influenza A(H1N1)pdm09 in Melbourne, Australia. Students and school settings were targeted for public health interventions following the emergence of pH1N1. This study was conducted to describe changes in social contacts among the earliest confirmed student cases of pH1N1 in Melbourne, Australia, to inform future pandemic control policy and explore transmission model assumptions.

**Methods:**

A retrospective cross-sectional behavioural study of student cases with laboratory-confirmed pH1N1 between 28 April and 3 June 2009 was conducted in 2009. Demographics, symptom onset dates and detailed information on regular and additional extracurricular activities were collected. Summary measures for activities were calculated, including median group size and median number of close contacts and attendance during the students’ exposure and infectious periods or during school closures. A multivariable model was used to assess associations between rates of participation in extracurricular activities and both school closures and students’ infectious periods.

**Results:**

Among 162 eligible cases, 99 students participated. Students reported social contact in both curricular and extra-curricular activities. Group size and total number of close contacts varied. While participation in activities decreased during the students’ infectious periods and during school closures, social contact was common during periods when isolation was advised and during school closures.

**Discussion:**

This study demonstrates the potential central role of young people in pandemic disease transmission given the level of non-adherence to prevention and control measures. These finding have public health implications for both informing modelling estimates of future pandemics and targeting prevention and control strategies to young people.

Initial reports of confirmed cases of pandemic influenza A(H1N1) 2009 (pH1N1) in Australia and internationally suggested that students comprised the majority of early cases. (*1*-*7*) This may have been due to numerous and prolonged contacts in classroom settings, heterogeneous mixing across age groups and both casual and sustained social contacts in non-school settings. (*8*-*12*) Consequently, students and school settings were targeted by a suite of public health interventions to contain community transmission during the immediate period following pH1N1 detection in Melbourne, the capital city of the Australian state of Victoria (population > 3.5 million). Such interventions included school closures, use of antiviral treatment and masks, isolation of cases and quarantine of contacts. (*13*, *14*)

An important driver of infectious disease transmission is the contact pattern and subsequent transmission of infection between and within groups of individuals, which may differ among different age groups. However, there is a lack of data for which key parameters, such as the number and frequency of contacts, as well as mixing between people according to age, can be estimated. (*8*, *12*, *15*) Further, decision-making about implementing pandemic influenza management plans are generally guided by mathematical models that compare the potential impact of prevention and control measures such as school closures, provided there is adequate information on the effect of these interventions on contact and transmission patterns within and across groups involved in the intervention. (*12*) In this study we collected empirical data to quantify social interactions of students and to describe changes in activity participation and social contacts following symptom onset and during school closures to inform future pandemic influenza policy and infectious disease transmission models assumptions.

## Methods

### Study design, recruitment and data collection

A retrospective cross-sectional behavioural survey was conducted. Eligible cases were students notified with laboratory-confirmed pH1N1 between 28 April and 3 June 2009 who attended primary or secondary schools in Melbourne, Australia with 10 or more confirmed cases notified during the same period. This period corresponded to the “Delay” (28 April to 21 May 2009) and “Contain” (22 May to 3 June 2009) phases of the Australian Health Management Plan for Pandemic Influenza. During these phases, the emphasis was on active case-finding and slowing community transmission of pandemic influenza through prevention and control measures. (*13*, *16*, *17*)

Cases were recruited by mail and telephone; up to five calls were attempted. Interviews were conducted either face to face at the students’ schools or households or by telephone between 18 November and 21 December 2009. Data collected, described in detail previously, (*18*) included demographic and case details, as well as specific information on social contacts between 11 May and 14 June 2009. This five-week period included all of the dates of symptom onset reported by the Victorian Department of Health and Human Services and was sufficient to capture activities during cases’ exposure and infectious periods.

Participants retrospectively completed a health diary that included information about their illness; the date of symptom onset, symptoms and measures taken to reduce symptoms or prevent transmission; their activities; and group contact. Written consent was obtained from each participant or their parent/guardian if the participant was younger than 18 years.

### Measures

Students were asked about their regular extracurricular activities, defined as regularly scheduled activities in addition to school. These included university classes (in Australia, high-achieving students can complete university studies alongside their final year of high school), part-time employment, sporting activities and religious groups. For each group or activity, students reported the number of social contacts (defined as the number of people in the group or activity), number of close contacts (defined as individuals within 1 m of a case for more than 15 minutes) and the dates that the group or activity took place. Students were also asked to describe additional extracurricular activities, such as social events, private classes (or example, one-on-one classes for music) or school social events.

From this, it was determined if students attended school or participated in extracurricular activities during their potential exposure period (defined as up to seven days before symptom onset), during their infectious period (defined as one day before symptom onset to seven days after symptom onset) or during the period of school closure (including weekends when school closures extended through a weekend).

### Data analysis

The mean number of groups and activities reported for each student, the median group or activity size and the number of close contacts per group or activity was calculated. The total number of close contacts per student was calculated by combining the number of unique close contacts at school, university, part-time employment, and sporting, religious and additional extracurricular activities for each individual.

A multivariable model using a generalized estimating equations regression was developed to assess associations between rates of participation in extracurricular activities and both school closures and the students’ infectious periods. The model used a negative binomial family function, a log link and an exchangeable within-participant correlation structure. The model was adjusted for school and potential interaction between the effect of school closures and infectious period. Statistical analyses were conducted using STATA version 15 (StataCorp, College Station, Texas, USA) and Microsoft Excel (Microsoft Corporation, Redmond, WA, USA).

### Ethical approval

Ethical approval was obtained from the Alfred Hospital Ethics Committee and Australian National University Ethics Committee.

## Results

There were seven schools in Victoria with more than 10 confirmed cases of pH1N1. The 162 case-patients from these schools were invited to participate; 99 (61%) were interviewed, 38 (24%) were not contactable and 25 (15%) refused or were not available to participate. Students that participated in the study were similar in age structure (*P* = 0.62) and in the schools attended (*P* = 0.42) to non-participants.

**Figure 1:**
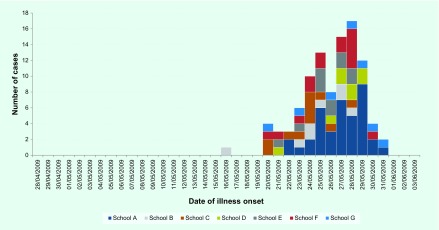
**Epidemic curve of the date of symptom onset for student cases of pH1N1 that were notified between 28 April 2009 and 3 June 2009 and participating in pH1N1 study, Melbourne, Australia**

Among the 99 respondents, there were more females than males (57% females). Half (49%) were in year 9 or year 10 (aged approximately 14–16 years) (**Table 1**). The earliest date of symptom onset was 16 May 2009 (this case was notified on 31 May 2009) (**Fig. 1**).

[**insert** Figure 1]

**Table 1:** Description of student cases of pH1N1 that were notified between 28 April 2009 and 3 June 2009 and participating in pH1N1 study, Melbourne, Australia

-	n	%
**Gender**		
Male	43	43
Female	56	57
**Age group**		
6–7 years	4	4
10–11 years	5	5
12–13 years	9	9
14–15 years	48	49
16–17 years	33	33
**School attended**		
School A	8	8
School B	8	8
School C	11	11
School D	8	8
School E	15	15
School F	11	11
School G	38	38
**Year level**		
Primary School	9	9
Year 7 (12–13 year olds)	5	5
Year 8 (13–14 year olds)	6	6
Year 9 (14–15 year olds)	25	25
Year 10 (15–16 year olds)	24	24
Year 11 (16–17 year olds)	13	13
Year 12 (17–18 year olds)	17	17

Note: percentages do not equal 100% due to rounding

Five of the seven schools closed in response to pH1N1, and the earliest date of school closure was 25 May 2009. The number of days that schools closed ranged from three to nine days (not including weekends).

Students reported that they regularly attended or participated in sports (*n* = 62), religious activities (*n* = 20), part-time employment (*n* = 18) and university classes (*n* = 10, **Table 2**). Among students that reported part-time employment, the most common workplaces were shops or department stores (*n* = 6, 33%), followed by supermarkets (*n* = 4, 22%), fast-food restaurants (*n* = 4, 22%) and cafes (*n* = 2, 11%). Among students that reported participating in sports (*n* = 62), the majority (*n* = 34, 55%) played in indoor settings while the rest played in outdoor settings (*n* = 27, 44%) or both (*n* = 1, 2%, data not presented in tables). The majority of students (*n* = 81, 81%) also reported additional extracurricular activities, including attending a school disco (*n* = 33, 41%), private classes (*n* = 11, 14%), school excursions (*n* = 8, 10%), school camps (*n* = 6, 7%), youth groups (*n* = 5, 6%) and a carnival (*n* = 2, 2%). Students reported varying levels of social contact in school and non-school settings. The median class size at school was 20 people. The median group size for non-school setting activities ranged from 12 (university class) to 175 (religious groups).

**Table 2:** Number of student cases of pH1N1 that were notified between 28 April 2009 and 3 June 2009 and participating in pH1N1 study that reported participation in school and extracurricular activities and groups and median group size

-	Regular activity	Median size of group or activity	Attended/participated in during potential exposure period		Attended/participated in during infectious period		Attended/participated in during school closure	
** -**	***n***	***n***	***n***	**%**	***n***	**%**	***n***	**%**
School	99	20	99	100%	98	99%	0	0%
University class	10	12	0	0%	0	0%	2	20%
Part-time work	18	20	0	0%	0	0%	2	11%
Sports	62	16	58	94%	28	45%	14	23%
Religious activity	20	175	20	100%	8	40%	1	5%
Other extra-curricular activity	81	30	81	100%	35	43%	21	26%

Note: The median size of the group relates to the total number of people in attendance at each specific class, group or activity. The median size of school group is based on the reported size of each class that students attended.

**Figure 2:**
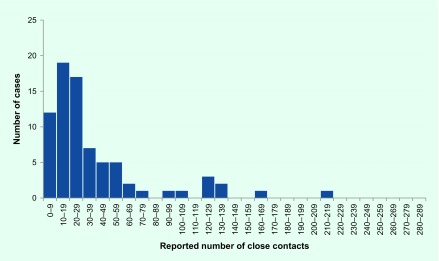
**Frequency of the total number of close contacts reported by student cases of pH1N1 that were notified between 28 April 2009 and 3 June 2009 and participating in pH1N1 study, Melbourne, Australia**

The median number of close contacts at school was three per class, and the median number of close contacts in non-school settings was similar, ranging from two (university class) to four (religious group, data not presented). The mean number of total close contacts was 45; distribution was highly dispersed and right tailed (**Fig. 2**).

[**insert** Figure 2]

Participation in groups and activities was less during school closures and during the students’ infectious periods compared to non-outbreak periods when schools were open and students were participating in regular activities. During their period of infectiousness, nearly all students attended school (*n* = 98, 99% of all students); however, no students attended university classes or work and there was reduced participation in sports (*n* = 28, 45% of the 62 students that regularly had sporting activities), religious (*n* = 8, 40%) and additional extracurricular activities (*n* = 35, 43%) (**Table 2**).

During school closures, there was less participation reported for sports (*n* = 14, 23% of the 62 students that regularly had sporting activities), religious (*n* = 1, 5%) and additional extracurricular activities (*n* = 21, 26%). Compared to non-outbreak periods, the incidence rate for participating in extracurricular activities was approximately one quarter during periods of school closures [incidence rate ratio (IRR) 0.28, 95% confidence interval (CI):0.17–0.46] and approximately one half during the students’ infectious periods (IRR 0.56, 95% CI:0.44–0.71, **Table 3**). There was no statistically significant interaction between the effect of infectious period and school closures.

**Table 3:** Adjusted incidence rate ratios for extra-curricular participation during students’ infectious periods and school closure periods, among 98 students with pH1N1 notification between 28 April 2009 and 3 June 2009

-	Adjusted incidence rate ratio	95% CI for adjusted incidence rate ratio	p-value
Infectious period	0.56	0.44–0.071	< 0.001
School closures	0.28	0.17–0.46	< 0.001

Note: Estimated using a multivariable generalized estimating equation model with a negative binomial family, log link and exchangeable correlation structure. In addition to infectious period and school closures, model was adjusted for school. Possible interactions between infectious period and school closures were assessed but were not statistically significant and were not included in the final model.

## Discussion

Several studies have demonstrated high transmission of pH1N1 in schools. (*7*, *11*, *19*-*23*) This study provides novel evidence of the potential of pH1N1 transmission within school and non-school settings via student networks and shows that students engaged in multiple activities in a range of settings during the pandemic period, even when public health interventions were implemented. While participation was less, students continued to engage in non-school-based activities during their periods of infectiousness and school closures.

The structure of Australian secondary schools, in which students move from class to class throughout a single school day, highlights how pandemic influenza can spread in school settings with relative ease. Additional school-based non-curricular activities observed in this study, such as sports groups, choir, excursions, carnivals and school camps, potentially interlink students across year levels, providing additional mechanisms for the transmission of pandemic influenza in young people.

There was a diverse range of social contacts in non-school settings reported by students. That just under one fifth of students reported engaging in regular part-time employment provides a risk factor for exposure of secondary transmission that has not previously been highlighted in studies that explore transmission of pH1N1. This employment resulted in varied social contacts in settings that involved numerous instances of both random and non-random social contacts (i.e. customers versus work colleagues) and included supermarkets, cafes and fast-food restaurants. While comparative data are not currently available to assess the differences in social contacts in workplace settings between teenagers and adults, these findings identify an important non-school setting for pH1N1 transmission for consideration in pandemic planning.

Similarly, information was captured on the level and type of sporting activities in which students engaged. That many students participated in sporting activities during their infectious period and during school closures is similar to that reported in Western Australia where sporting activities were commonly reported by students (cases and non-cases) over a longer period in 2009. This study also found that many team sports were played in an indoor setting, providing opportunities for disease transmission. (*6*)

Social distancing recommendations, such as the isolation of cases during their infectious period, were poorly adhered to by our sample. Students reported high levels of school attendance after symptom onset and while potentially infectious, thus further contributing to the evidence that schools are effective settings for the spread of pandemic influenza. Anecdotal evidence from some students suggested they did not want to be absent from school because of senior-school examinations during the time period. While this provides some explanation for the high level of school attendance, it nonetheless highlights the need for improved communication at the individual level to prevent community transmission. This communication should be aimed at social isolation of symptomatic cases, including while schools remain open and pandemic influenza is potentially circulating within schools.

The participation levels of students in sporting, religious and additional extracurricular activities in the week following symptom onset and while potentially infectious decreased compared to the levels reported as a regular activity. While somewhat helpful, decreased attendance does not meet isolation recommendations during the potentially infectious period. This reduced participation is likely influenced by the presence of symptoms among the samples and possibly because some students were undertaking examinations at this time. Participation in activities, especially while symptomatic, could potentiate transmission within and across social groups and hence be a bridge between young people and the wider community. Other international studies have also documented that social events such as parties and religious activities were implicated in transmission of pH1N1. (*5*, *6*) This reinforces the need for improved communication regarding social isolation to include extracurricular groups and activities to maximize the effect of social distancing measures in controlling pandemic influenza.

There was also lower participation in sporting, religious and additional extracurricular activities during school closures. This is similar to a study that compared the social contact patterns of students (pH1N1 cases and non-cases) before and after school closure that found that fewer students visited public places (such as shops, places of worship and playing fields) when school was closed than when open. (*22*) However, in the Western Australian study, it was reported that almost three quarters of students (influenza cases and non-cases) left home at least once during school closures. (*6*) This finding reinforces the need for strategies in the revised pandemic plan to ensure that the benefit of school closures – that is, reduced social contact between students – is realized and to prevent students’ social contact with potentially broader and unexposed social networks.

The distribution of the total number of close contacts reported by students was highly dispersed and was skewed to the right with the majority of students having a small number of close contacts and a few having much larger numbers of contacts. This has ramifications for the control of disease spread, as containment is more difficult than for a random network of contact between people. Targeted strategies aimed at those more central to the network or with a greater number of social ties may be more efficient than non-targeted strategies. Although impractical to target individuals with many contacts, it may be possible to identify and target activities that lead to the skewed distribution such as religious gatherings or large gatherings.

This study has limitations, some of which have been documented previously, (*18*) including issues relating to possible selection and recall bias. In addition, the number of social contacts reported here are likely to be an underestimation given that questions were asked about specific planned activities rather than incidental activities and that information was collected retrospectively. Future research to enumerate interactions that are not class or group based would fill this gap in information. Further, the number of contacts in this study was measured by recalling close contacts over a 35-day period, rather than daily, which is the norm in studies of social contacts. (*12*, *24*) The relationship between contact ties and interactions is an emerging area of social network research and is likely to be a key determinant in infectious disease transmission. (*25*)

The results from this study have public health implications for both informing modelling estimates of future pandemics and targeting prevention and control strategies to young people. School closures can only prevent transmission between students that could occur at school or school-based activities such as school camps. Young people participate in numerous activities outside of school hours and continue to engage with other young people via additional extracurricular activities during school closures. This study also identified the possibility of targeted strategies for transmission prevention given the highly dispersed nature of students’ contact networks. Young people are not a homogenous group and may play a central role in future influenza pandemics. Therefore it is critical that any response to pandemic influenza considers the mechanisms of transmission through young people.
